# Enzymatic Functions for Toll/Interleukin-1 Receptor Domain Proteins in the Plant Immune System

**DOI:** 10.3389/fgene.2020.00539

**Published:** 2020-06-02

**Authors:** Adam M. Bayless, Marc T. Nishimura

**Affiliations:** Department of Biology, Colorado State University, Fort Collins, CO, United States

**Keywords:** Toll/interleukin-1 receptor, TIR, NLR, NADase, innate immunity

## Abstract

Rationally engineered improvements to crop plants will be needed to keep pace with increasing demands placed on agricultural systems by population growth and climate change. Engineering of plant immune systems provides an opportunity to increase yields by limiting losses to pathogens. Intracellular immune receptors are commonly used as agricultural disease resistance traits. Despite their importance, how intracellular immune receptors confer disease resistance is still unknown. One major class of immune receptors in dicots contains a Toll/Interleukin-1 Receptor (TIR) domain. The mechanisms of TIR-containing proteins during plant immunity have remained elusive. The TIR domain is an ancient module found in archaeal, bacterial and eukaryotic proteins. In animals, TIR domains serve a structural role by generating innate immune signaling complexes. The unusual animal TIR-protein, SARM1, was recently discovered to function instead as an enzyme that depletes cellular NAD^+^ (nicotinamide adenine dinucleotide) to trigger axonal cell death. Two recent reports have found that plant TIR proteins also have the ability to cleave NAD^+^. This presents a new paradigm from which to consider how plant TIR immune receptors function. Here, we will review recent reports of the structure and function of TIR-domain containing proteins. Intriguingly, it appears that TIR proteins in all kingdoms may use similar enzymatic mechanisms in a variety of cell death and immune pathways. We will also discuss TIR structure–function hypotheses in light of the recent publication of the ZAR1 resistosome structure. Finally, we will explore the evolutionary context of plant TIR-containing proteins and their downstream signaling components across phylogenies and the functional implications of these findings.

## The Plant Immune System

**FIGURE 1:**
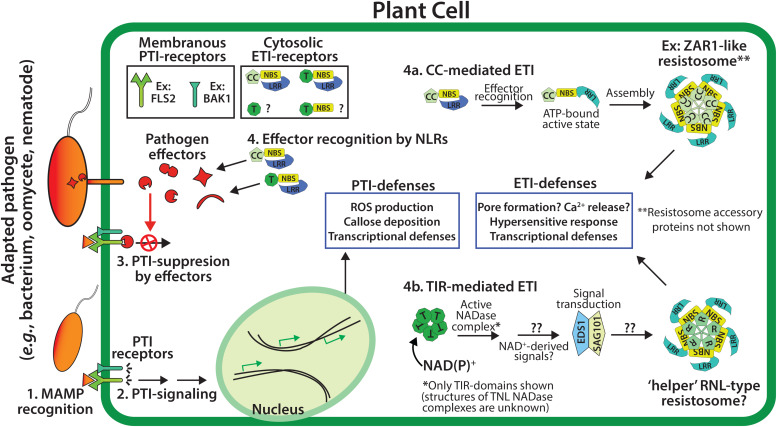
Overview of the two tiers of the plant immune system. PTI (pattern-triggered immunity) utilizes membrane associated PTI receptors to detect conserved microbial associated molecular patterns (MAMPs, e.g., chitin or flagellin), and signal downstream PTI-immunity **(steps 1 and 2)**. The effectors of adapted pathogens can disarm PTI-immunity **(step 3)**, while genetically resistant plants utilize NLR-type (NBS-LRR) resistance proteins to detect effector activities and trigger ETI (effector-triggered immunity, **step 4**), which often includes localized cell death - the hypersensitive response (HR). Plant NLR resistance proteins generally possess N-terminal CC (coiled-coil) or TIR (Toll/interleukin-1 Receptor) domains.

Single and multicellular organisms have evolved numerous defenses to ward off biotic challenges. The plant innate immune system consists of receptor proteins that monitor both extracellular and intracellular pathogen-related signals to activate defenses (Figure 1). Typically, extracellular signals are transduced across the plasma membrane by an extensive array of receptor-like kinase (RLK) and receptor-like proteins (RLPs) (Boutrot and Zipfel, 2017; Tang et al., 2017). Disease resistance conferred by the RLK/RLP pattern recognition receptor (PRR) system is triggered by a wide array of apoplastic molecules from microbes, pathogens and host damage signals. Accordingly, pathogens have evolved to extensively target PRR pathways to promote host susceptibility. A common strategy of plant pathogens is to deliver intracellular virulence proteins (such as type III effector proteins) in order to disrupt PRR-based defense (Jones and Dangl, 2006; Dangl et al., 2013). These virulence proteins are necessary for pathogenicity, and thus serve as reliable indicators of pathogen presence. In response to pathogen immunosuppression, plants have evolved a second layer of innate immune receptors that directly or indirectly recognize the presence of pathogen virulence proteins (Jones and Dangl, 2006; Qi and Innes, 2013). As such, virulence proteins are the tools that pathogens use to suppress the host immune system, but also the signals that plants of the correct genotype (i.e., resistant plants) can recognize to reinitiate a defense response. These intracellular receptors are characterized by nucleotide-binding site (NBS) domains and a C-terminal Leucine-rich repeat (LRR). This combination of domains is present in both plant and animal NLR proteins (confusingly referring to both “NBS-LRR” and “Nod-like receptors (Nod: N-terminal oligomerization domain).” While plant and animal NLR proteins are functionally conserved in many ways, it appears that they are the product of convergent evolution (Urbach and Ausubel, 2017).

The recognition of intracellular pathogen virulence molecules promotes conformational changes in NLR proteins (Takken and Goverse, 2012). The N-terminal domain of NLR proteins has signaling activities, while the C-terminal NBS-LRR domains negatively regulate signaling in the resting state. The NBS domain functions as a molecular switch depending on the bound nucleotide: ADP-bound in the resting state and ATP-bound in the active state (Takken and Goverse, 2012). Both plant and animal NLRs are auto-regulated and self-associate during signal transduction, however, the N-terminal signaling domains of plant and animal NLRs are distinct (Qi and Innes, 2013; Hu et al., 2015; Nanson et al., 2019). Generally, plant NLRs contain N-terminal TIR (Toll/Interleukin Receptor-1) or CC (coiled coil) domains, and are therefore known as TNLs or CNLs (Qi and Innes, 2013). Monocot genomes appear to lack TNL loci, however, both monocots and dicots can encode TIR-only and TIR-NBS proteins (Meyers et al., 2002; Collier et al., 2011; Nandety et al., 2013; Nishimura et al., 2017; Gao et al., 2018). TIR and CC-domains from plant NLRs are sufficient to activate immune outputs, including a localized cell death termed the hypersensitive response (HR), and transcriptional defense programs (Swiderski et al., 2009; Collier et al., 2011). The self-association and oligomerization of either TIR or CC-domains is required for plant immune signaling, however, the downstream events which follow the activation of TIR or CC resistance proteins has remained unclear (Casey et al., 2016; Wan et al., 2019; Wang et al., 2019a).

## Downstream Components of TIR-Signaling Pathways in Plants

**FIGURE 2:**
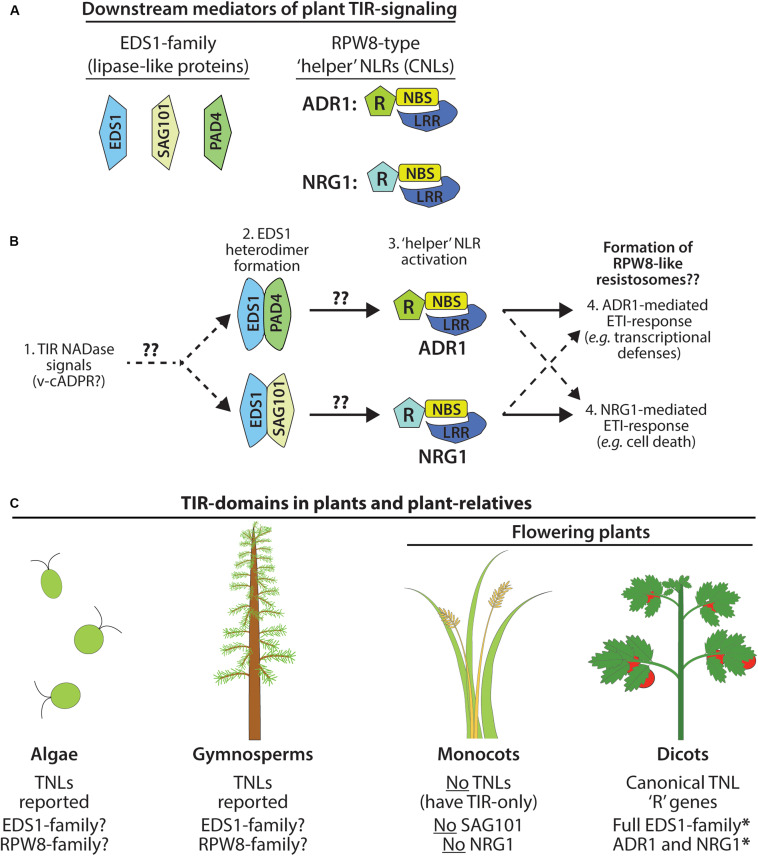
Immune signaling by plant TIR NADases requires downstream components. **(A)** Members of the EDS1 lipase-like family: EDS1, SAG101, PAD4. Terminal mediators of TIR-signal transduction are the RPW8-type ‘helper’ RNLs: ADR1 and/or NRG1. **(B)** Model of ETI-pathway activation by plant TIR NADases. Perception of plant TIR signals (e.g., v-cADPR?) promotes EDS1-family heterodimerization, and subsequent activation of the ‘helper’ RNLs, ADR1 or NRG1. EDS1-PAD4 heterodimers may favor activation of ADR1-mediated responses (transcriptional defense programs), while EDS1-SAG101 heterodimers activate NRG1-mediated responses (cell death). Functional redundancy among NRG1 and ADR1 indicated by dashed arrows. **(C)** TIR-domain containing proteins, including TNLs, are found in the genomes of phylogenetically distant plant-lineages and in the relatives of land plants, including green algae (Sun et al., 2014; Shao et al., 2019), as well as gymnosperms (western white pine) and the moss, *Physcomitrella patens*. Monocots do not encode TNLs and lack two downstream mediators of TIR-immune signaling: SAG101 and NRG (Collier et al., 2011; Lapin et al., 2019).

Genetic screens have identified two families of proteins that appear universally required for plant TIR phenotypes (Figure 2). The first component is the EDS1 (Enhanced Disease Susceptibility 1) family of lipase-like proteins [EDS1, SAG101 (Senescence-Associated Gene 101), and PAD4 (Phytoalexin Deficient 4)] (Feys et al., 2005; Lapin et al., 2019). The second component, the RPW8 class of ‘helper’ CNLs, often referred to as ‘RNLs,’ functions downstream of the EDS1 family (Peart et al., 2005; Qi et al., 2018; Jubic et al., 2019; Wu et al., 2019). Helper NLRs such as the NRG1 (N-requirement Gene 1) and the ADR1 family (Activated Disease Resistance 1) are candidates for being the ultimate output of TIR pathways (Collier et al., 2011; Qi et al., 2018; Jubic et al., 2019). How these downstream components are activated by TIR oligomerization, and the organization of the overall pathway, remains a major unanswered question (Jubic et al., 2019; Lapin et al., 2019; Wan et al., 2019).

EDS1 forms exclusive heterodimers with either PAD4 or SAG101 to relay TIR-immune signals (Feys et al., 2005; Wagner et al., 2013). EDS1 and PAD4 are also reported to function in plant basal defenses and salicylic acid signaling (Cui et al., 2017). The crystal structure of the EDS1-SAG101 heterodimer suggests that binding of the N-terminal lipase-like domains establishes unique interaction interfaces at the C-terminal EP domain (Wagner et al., 2013). The C-terminal EP-domain of EDS1-members contains positively charged residues and is essential for transduction of TIR-signals (Bhandari et al., 2019; Lapin et al., 2019). The TNL RPS4 (Resistance to *Pseudomonas syringae* 4), as well as particular TIR-NBS proteins, have been reported to associate with EDS1, as has the ‘helper’ NLR, NRG1 (Heidrich et al., 2011; Nandety et al., 2013; Huh et al., 2017; Qi et al., 2018). The functional consequences of these physical interactions are unknown. Lapin et al. (2019) determined that the EDS1-members of *Solanaceous* species could complement a *N. benthamiana* mutant which lacks all EDS1-family members. However, the orthologous EDS1-members of *Arabidopsis* did not complement, suggesting that within species, EDS1-members may have co-evolved a high degree of specificity in the relay of TIR-signals (Lapin et al., 2019). Curiously, in the absence of downstream ‘helper’ NLRs, EDS1-members can still mediate limited transcriptional defense programs from an auto-active version of the TNL, Roq1 (Recognition of XopQ1) (Qi et al., 2018).

The expression of the RPW8-domains of ADR1 or NRG1 is sufficient to trigger HR, even in *eds1* null backgrounds, placing ‘helper’ RNLs as downstream mediators of TIR-signaling (Collier et al., 2011; Qi et al., 2018). Typically, plant genomes carry relatively few loci encoding helper RNLs, consistent with a conserved RNL function that integrates inputs channeled from upstream TNL receptors via EDS1-complexes. Additionally, functional redundancy between ADR1 and NRG1 has been reported (Castel et al., 2019; Jubic et al., 2019; Lapin et al., 2019; Wu et al., 2019). Some CNLs are also reported to signal through ADR1, suggesting that cross-talk might occur at the endpoints of certain CNL and TNL-signal pathways (Castel et al., 2019; Wu et al., 2019). The RPW8-domain of helper RNLs does share similarities with the CC-domain of CNLs; thus, the recent structure of the ZAR1 (HOPZ-ACTIVATED RESISTANCE 1, a CNL) resistosome may provide insights into the functions of the ADR1 and NRG1 helper NLRs (Wang et al., 2019a, b). The active ZAR1 complex assembles into a ring-shaped pentamer, the “resistosome,” and hypothetically disrupts cell membrane integrity with a pore-forming channel (Wang et al., 2019a, b).

**FIGURE 3:**
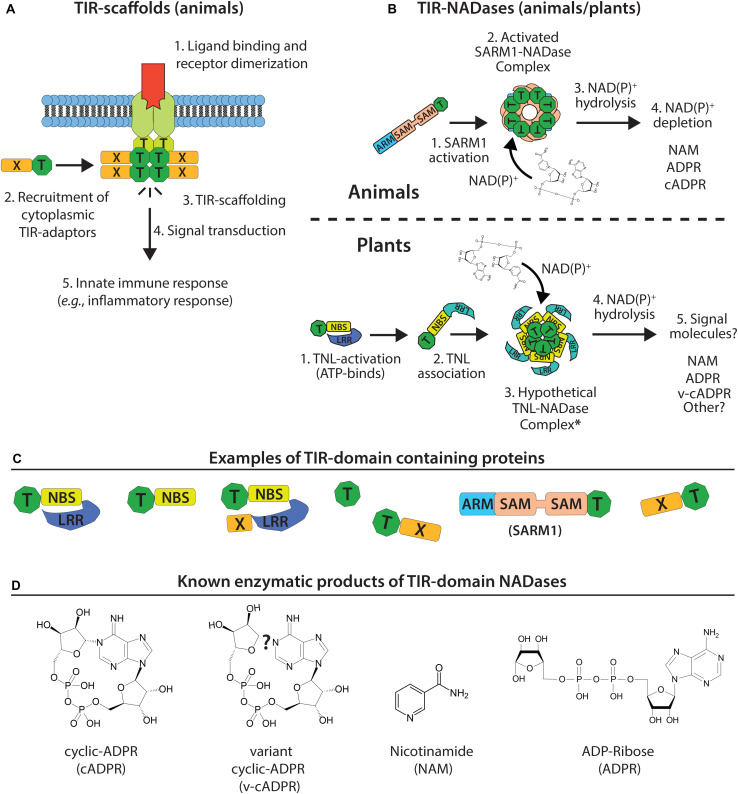
Model of TIR-domain scaffolding (animals) and TIR-NADase activity (plants and animals). **(A)** Canonical TIR-scaffold function in animals: TIR-TIR interactions promote signal complex formation and innate immune signal transduction. **(B)** Top: animal TIR NADases (e.g., SARM1) assemble into high order complexes, and hydrolyze NAD(P)^+^ substrate and alter NAD(P)^+^ pools. Bottom: assembly of plant TIR-domains into hypothetical NADase complex (resistosome-like?) and generation of immunomodulatory signals. **(C)** Numerous TIR-domain configurations are present in animal, plant, and bacterial proteins. Plant TIR-domains are often found in modular NBS-LRRs, TIR-NBS, TIR-X or TIR-only proteins. -X corresponds to atypical or undefined domains. The animal SARM1 TIR is located at the C-terminus; the SARM1 SAM-domains promote oligomerization. **(D)** Known products of TIR NADases; plant TIRs produce variant cyclic-ADPR (v-cADPR), whose structure is currently unknown.

The mechanisms of how plant NLRs activate downstream immunity is an active area of research. While TIR–TIR interactions are well known to promote animal immune signaling via scaffold function, a new paradigm of plant TIR function has recently emerged: signal competent plant TIR-domains are NAD^+^-(nicotinamide adenine dinucleotide)-hydrolyzing enzymes (Figures 3A–D) (Horsefield et al., 2019; Wan et al., 2019). Below, we review recent advances in the understanding of plant TIR-domain structure, evolution, and enzymatic (NADase) function. We also draw insights from the TIR-NADases encoded by animals and prokaryotes, and explore how the newly reported structure of the ZAR1 CNL ‘resistosome’ complex might inform the high order complexes of plant TIR-NADases.

## TIR-Domains: a Cellular Defense Module Found in All Domains of Life

**FIGURE 4:**
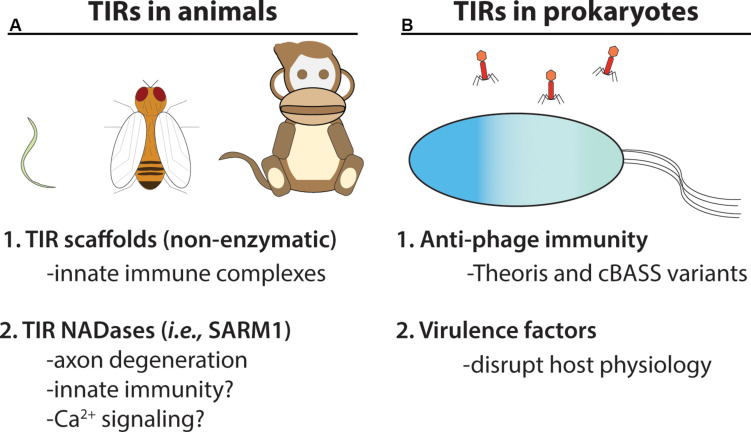
TIR NADases were recently reported in animals and prokaryotes. **(A)** Diverse eukaryotic organisms, including invertebrates (e.g., *C. elegans, D. melanogaster*) and vertebrates, utilize TIR-domain containing proteins in cellular innate immunity. Non-enzymatic TIR-domain containing proteins in animals promote signal complex formation via TIR – TIR interactions. The SARM1 NADase TIR from animals functions in axon degeneration, and is reported to function in immunity in *C. elegans* (Shivers et al., 2009). **(B)** Numerous bacteria and archaeal species encode TIR-NADases. Prokaryotic TIR-domain containing proteins are reported to function in anti-phage immunity (Thoeris system and variants of cBASS) (Doron et al., 2018; Cohen et al., 2019). TIR-domains from pathogenic bacteria are reported to function in virulence (Alaidarous et al., 2014).

Toll/Interleukin Receptor-1 (TIR)-domain containing proteins are found in all domains of life – Eukarya, Bacteria, and Archaea (Figures 4A,B) (Essuman et al., 2018). Frequently, TIR-domain containing proteins function in immunity or cell death decisions in bacteria, plants and animals, suggesting an ancient role in cellular defenses (Figures 3A,B, 4A,B). The core TIR-domain is typically ∼120–200 residues, and is found in multi-domain and single domain proteins (Nimma et al., 2017). TIR-domains generally require TIR-TIR self-associations for function, and TIR-domains can also participate in heterotypic protein interactions. The sequence identity of TIR-domains among different species may be as low as 20–30%, however, TIR-domains share a flavodoxin-like fold, consisting of parallel beta-sheets and alpha-helices with interconnecting loops (Ve et al., 2015).

## Insights to Plant TIR Function From Animal Systems: SARM1 (Sterile Alpha and TIR Motif-Containing 1) Is an NADase

Typically, animal TIRs (e.g., Toll-like receptors, MyD88) couple pathogen detection to defense gene activation by nucleating the formation of large multimeric signaling complexes (Figure 3A) (Xu et al., 2000; O’Neill and Bowie, 2007; Kenny and O’Neill, 2008; Nimma et al., 2017). Crystal structures for numerous animal TIR-domains have acted as guides for a biochemical dissection of TIR-domain function (Xu et al., 2000; Valkov et al., 2011; Bovijn et al., 2012). The crystal structure of the TIR-domain from Toll Like Receptor 2 (TLR2) revealed residues required for TIR-TIR interactions, and the core TIR-domain structure of parallel beta-sheets and alpha-helices (Xu et al., 2000). Additional structural studies of TIR-adaptor proteins further defined TIR interfaces required for multimerization and signal complex formation (Nyman et al., 2008; Valkov et al., 2011; Bovijn et al., 2012). Animal TIR scaffolding can signal various defensive outputs, such as inflammatory responses and cytokine production (Figure 2A) (O’Neill and Bowie, 2007). In contrast, the unusual animal TIR protein SARM1 (*sterile alpha and TIR motif-containing 1*) was recently found to have a surprising enzymatic function (Essuman et al., 2017).

The animal TIR protein SARM1 functions in axon degeneration, an active process of programmed cell death in response to injury (classically known as “Wallerian degeneration”) (Gerdts et al., 2015). NAD^+^-depletion had been associated with axon degeneration, but the SARM1-regulated NADase had remained elusive. The critical observation that the TIR domain of SARM1 is structurally similar to bacterial nucleotide-processing enzymes led to the recognition that the SARM1 TIR has an intrinsic enzymatic activity: NAD^+^-hydrolase function (Figure 3B) (Gerdts et al., 2015; Essuman et al., 2017). Axon degeneration requires SARM1 TIR domain NADase activities (Essuman et al., 2017). The unusual enzymatic activity of SARM1 TIR relative to other animal TIR domains is perhaps reflected in an unusual evolutionary history, as the SARM1 TIR appears to have been horizontally transferred into animals (Zhang et al., 2011). TIRs that function in canonical TLR pathways (TLR4 and MyD88) do not have NADase activity, although the family has not been exhaustively tested (Essuman et al., 2017).

Like NLRs, SARM1 is a multidomain TIR protein that is auto-inhibited. SARM1 has two tandem sterile alpha (SAM) domains, which enable oligomerization, and an N-terminal Armadillo domain, which is required for auto-inhibition (Figures 3B,C) (Essuman et al., 2018). SARM1 TIR NADase function is dependent upon oligomerization and TIR-TIR associations. The mechanism of activation during axon degeneration is unclear, but NADase activity of SARM1 can be enhanced by phosphorylation or treatment with a cell-permeant mimetic of nicotinamide mononucleotide, an NAD^+^ precursor (Murata et al., 2018; Zhao et al., 2019).

NAD^+^-hydrolysis by SARM1 generates ADPR (ADP-ribose), cyclic ADPR (c-ADPR) and NAM (nicotinamide) (Essuman et al., 2017) (see Figure 3D). The products of SARM1-mediated NAD^+^-hydrolysis (cADPR, ADPR) are known Ca^2+^ mobilization agents and may thus effect cellular Ca^2+^ signaling (Lee, 2012; Guse, 2015; Lee and Zhao, 2019; Zhao et al., 2019). SARM1 readily hydrolyzes NADP^+^ as well as NAD-analogs with substitutions to the adenine ring, such as amino group additions (Essuman et al., 2017). However, FAD (flavin adenine dinucleotide) and NADH or NAD-analogs lacking the amino group of the nicotinamide ring could not be hydrolyzed (Essuman et al., 2017, 2018). Depending on local cellular pH, SARM1 is also reported to generate NAAD (nicotinic acid adenine dinucleotide) (Zhao et al., 2019).

**FIGURE 5:**
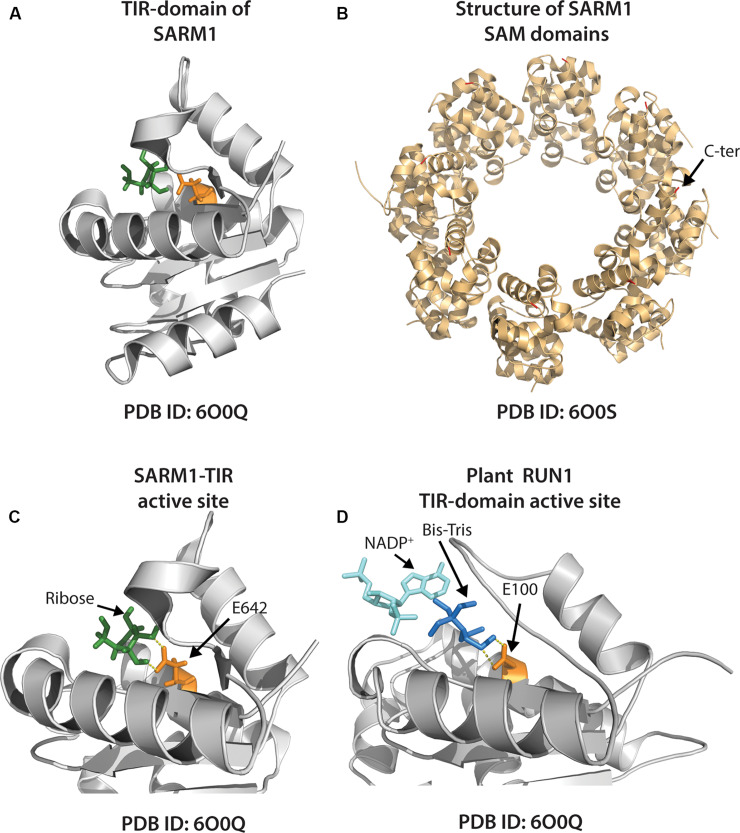
Structures of individual animal and plant TIR-domain NADases, and the higher order SARM1 SAM octamer. **(A)** Crystal structure of the SARM1-TIR domain (PDB ID: 6O0Q) with ribose (shown green) positioned near putative catalytic glutamate residue (E642), colored orange. **(B)** Crystal structure of tandem SAM domains of the animal TIR-NADase, SARM1 (PDB ID: 6O0S). The SARM1 SAM domains adopt a closed octameric ring conformation. C-terminal end of SAM tipped with red (arrow shown for one unit). **(C)** Close-up view of SARM1 TIR active site, as in **(A)**. Arrows indicate ribose and putative catalytic E642 (ribose ∼2.6Å from E642). **(D)** Close-up of active site of the TIR-domain from plant TNL, RUN1 (PDB ID: 6O0W). A bis-Tris molecule (dark blue) positioned near putative catalytic glutamate (E100, orange) precludes access of NADP^+^-substrate (aqua); bis-Tris ∼3 Å from E100.

A recent crystal structure of the SARM1 TIR reveals conservation with both plant and prokaryotic TIR-domains (Horsefield et al., 2019). The active site of the SARM TIR-domain includes a conserved glutamic acid (E642) which is required for NAD^+^-hydrolysis (Figure 5A). Recent crystal and cryo-EM structures of SARM1 complexes, and of the tandem SAM-domains, indicate that the active SARM1 NADase complex forms a ring-shaped octamer (Horsefield et al., 2019; Sporny et al., 2019) (Figure 5B). The crystal structure of the SARM1 TIR active site revealed close proximity of ribose with the putative catalytic glutamate (E642) and may suggest potential substrate-active site interactions (Figure 5C) (Horsefield et al., 2019). The exact catalytic mechanism of SARM1 is unknown, but appears distinct from CD38, which also produces cADPR from NAD^+^ (Loring et al., 2020).

Strikingly, SARM1 triggers cell death when transiently expressed in the leaves of the plant, *Nicotiana benthamiana* (Horsefield et al., 2019; Wan et al., 2019). Like axon degeneration, plant cell death triggered by SARM1 requires NADase function, however, SARM1-mediated cell death occurs independently of the known plant TIR-signaling components EDS1 and NRG1 (Horsefield et al., 2019; Wan et al., 2019). Notably, supplementation of exogenous NAD^+^ reduces axon degeneration mediated by SARM1 (Gerdts et al., 2015). As such, SARM1 depletion of cellular NAD(P)^+^ is likely to underlie both animal axon degeneration and plant cell death resulting from its ectopic expression. However, some cell lines are reported to tolerate low levels of SARM1 expression (Lee and Zhao, 2019; Zhao et al., 2019). Whether low level SARM1 activity in particular cellular contexts might generate signaling molecules vs. deplete cellular NAD^+^ stores, is not yet clear.

## TIR NADases in Prokaryotes: Phage Immune Systems and Virulence Factors

Numerous bacterial and archaeal species encode TIR-domain containing proteins, primarily of unknown function (Spear et al., 2009; Doron et al., 2018; Essuman et al., 2018). However, some prokaryotic TIRs are reported to function in anti-phage immunity, while other TIRs may act as virulence factors which manipulate host responses (Figure 4B) (Alaidarous et al., 2014; Doron et al., 2018; Coronas-Serna et al., 2019). TIR-domains encoded by *Brucella* and *Paracoccus* are reported to mimic animal TIR-adaptors and disrupt TLR immune signaling, potentially via physical interactions with animal TIR domains (Chan et al., 2009; Alaidarous et al., 2014; Snyder et al., 2014). However, many apparently non-pathogenic bacteria encode TIR-proteins, suggesting that some TIR-domains could possess functions outside of virulence or immunity (Spear et al., 2009). NAD^+^-hydrolase activities have recently been shown for several bacterial and archaeal TIRs, and thus, it has been suggested that ancestrally, the TIR-domain belongs to a large family of nucleotide hydrolase enzymes (Essuman et al., 2018).

Like the SARM1 TIR NADase, all examined prokaryotic TIRs also require the putative catalytic glutamate for NADase function (Essuman et al., 2018). Prokaryotic TIRs are likely to also require TIR-TIR self-associations, as local crowding (via TIR protein laden beads) enhanced NADase function (Essuman et al., 2018). Prokaryotic TIR-domains show variation in terms of NAD^+^-hydrolysis kinetics, as well as in the type and ratio of products produced from NAD^+^-hydrolysis (Essuman et al., 2017). For example, the TirS TIR domain from *Staphylococcus aureus* generated ADPR and cADPR, while the TcpO TIR domain from the archaea *Methanobrevibacter olleyae* produced a novel product initially termed metabolite X, which is likely a variant of cyclic ADPR (v-cADPR), whose structure remains unresolved (Essuman et al., 2017; Wan et al., 2019).

**FIGURE 6:**
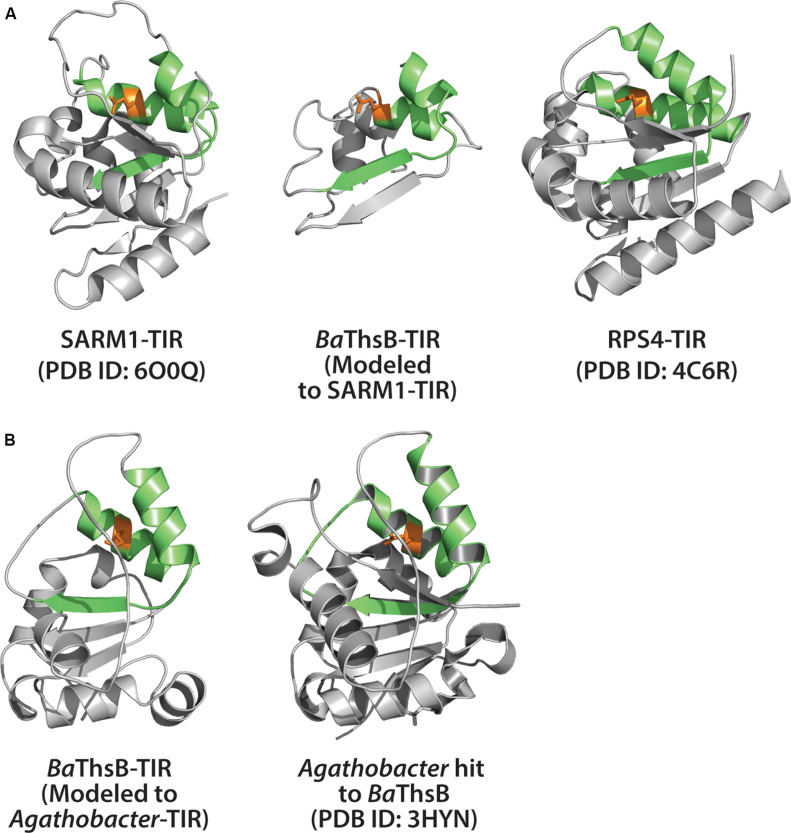
Structural modeling of ThsB (Thoeris TIR) from *Bacillus amyloliquefaciens* (*Ba*). **(A)** Center: Phyre2 modeling of *Ba*ThsB TIR-domain to the SARM1-TIR structure. Phyre2 model confidence: 96.2%; *Ba*ThsB-TIR amino acid identity to SARM1-TIR: 15%. Left: Alignment of SARM1-TIR (PDB ID: 6O0Q) to *Ba*ThsB-TIR. Right: Alignment of RPS4-TIR structure (PDB ID: 4C6R) to *Ba*ThsB-TIR (and to SARM1-TIR). **(B)** Phyre2 modeling of full length *Ba*ThsB to a putative signal transduction protein from *Agathobacter rectales* (a putative Thoeris system ThsB TIR). Phyre2 model confidence: 100%; *Ba*ThsB amino acid identity to *Agathobacter* ThsB match: 59%.

Recent studies from the Sorek lab may provide a glimpse into the origins of TIR-mediated immunity (Figure 4B) (Doron et al., 2018; Cohen et al., 2019). A survey of tens of thousands of prokaryotic genomes, coupled with functional screening, unveiled multiple new classes of anti-phage defense systems. Among these, an anti-phage system termed Thoeris, was found in ∼2,000 bacterial and archaeal genomes (Doron et al., 2018). The Thoeris defense operon encodes an NAD^+^ binding protein (ThsA) and a TIR-domain protein (ThsB). Both ThsA and B are required for anti-phage immunity. Amino acid alignment of the ThsB TIR-domain with the SARM1-TIR indicated conservation of the catalytic glutamate (Doron et al., 2018). We used Phyre2 to model the *B. amyloliquefaciens* encoded ThsB (*Ba*ThsB), and retrieved a top-match (60% identity, 100% confidence) to the crystal structure (PDB ID: 3HYN) of a putative signal transduction factor from *Agathobacter rectalis* (Figures 6A,B). A comparison of the SARM1 TIR and plant RPS4 TIR structures with the *Ba*ThsB TIR-domain model indicates positional conservation of the putative catalytic glutamate (Figure 6A). The putative catalytic glutamate (E99) of ThsB was required for phage protection, suggesting that TIR domains may have an ancient enzymatic-based immune function (Doron et al., 2018). It will be interesting to assess if Thoeris functions via NAD^+^-depletion, akin to SARM1, or could generate NAD^+^-derived immunomodulatory signals.

The Sorek group further reported that some prokaryote genomes harbor an ortholog of the cGAS-STING defense system found in animals (Cohen et al., 2019). Upon detecting invading DNA, cGAS (cyclic GMP-AMP synthase) generates cyclic GMP-AMP (cGAMP) via oligonucleotide cyclase activity. The cGAMP signal then promotes host cell demise through activating a phospholipase which disrupts membrane integrity (Cohen et al., 2019). This prokaryotic system was dubbed CBASS for cyclic oligonucleotide-based anti-phage signaling system. Notably, variants of CBASS-mediated immunity can encode TIR-domains (Cohen et al., 2019). Whether the TIR-domains of particular CBASS variants require NADase function is uncertain. Nonetheless, it is becoming clear that TIR-mediated immunity to phages is common in both bacteria and archaea. CBASS and Thoeris appear to trigger host cell death prior to the completion of viral replication, thereby restricting phage release into the bacterial population. Elucidating the molecular mechanisms of these prokaryotic TIR-based systems may provide insights into the evolution and function of both immunity and cell death in plants and animals.

## TIR NADase Activity in Plants

Similar to animal SARM1, plant TIRs were recently demonstrated to be NAD^+^ hydrolases, and this NADase activity is required to relay immune signals (Horsefield et al., 2019; Wan et al., 2019). Sequence analysis of the TIR-domain encoding genes from Arabidopsis, as well as ∼8,000 TIR sequences found from 108 available plant genomes, indicates high conservation (∼90%) of the putative catalytic glutamate required for NADase activity (Wan et al., 2019). The minority of TIR-domains that lack this conserved glutamate appear to be from ‘sensor-type’ TNLs which function via a signal-competent, genomically paired TNL. These sensor-type TNLs lack the ability to trigger cell death or immunity without their partner TNL (Wan et al., 2019).

Like SARM1 of animals, the NADase activity of plant TIRs was required for TIR-domain function; i.e., to relay immune signals (Horsefield et al., 2019; Wan et al., 2019). *In vitro* NADase cleavage activity was demonstrated by TIR-domains from full length TNLs, as well as TIR-only proteins from dicot plants (Horsefield et al., 2019; Wan et al., 2019). Similar to SARM1 TIR and prokaryotic TIRs, plant TIR-domains could utilize NAD^+^ and NADP^+^ as a substrate, but not the structurally related NAD^+^ precursor NAAD (nicotinic acid adenine dinucleotide) (Essuman et al., 2018; Horsefield et al., 2019; Wan et al., 2019). Intriguingly, a TIR-only protein from the monocot, *Brachypodium distachyon* (BdTIR), also displayed NAD^+^-hydrolysis, in addition to triggering an EDS1/NRG1-dependent HR, suggesting that TIR-immune signaling may be conserved among dicot and monocot plants (Wan et al., 2019). The products generated by plant TIR NADase reactions include NAM, ADPR, and v-cADPR. Unlike the SARM1 TIR, production of cyclic-ADPR by plant TIRs was not detected. v-cADPR has a near identical HPLC retention time and molecular mass to the product of an archaeal TIR, TcpO (Essuman et al., 2018; Wan et al., 2019).

A crystal structure of the plant TIR-domain, RUN1, with bound NADP^+^ substrate was determined by Horsefield et al. (2019) (Figure 5D). The putative catalytic glutamate of RUN1 was associated with a molecule of bis-Tris, while NADP^+^ was bound near the periphery of the proposed active site (Figure 5D). Accordingly, bis-Tris addition to RUN1 NADase assays inhibited activity, suggesting that bis-Tris association with active site residues may preclude NADP^+^ access and subsequent hydrolysis (Horsefield et al., 2019). How the NAD(P)^+^ substrate interacts with and positions in the active site of plant TIRs during catalysis remains to be determined.

## Plant TIR-Domain Self-Association Is Necessary for NADase Activity

Plant TIR-TIR self-association occurs through at least two known interfaces formed by pairs of alpha helices (denoted as ‘α’) (Bernoux et al., 2011; Williams et al., 2014, 2016). Both AE- (i.e., the αA/αE surface) and DE-type (αD/αE surface) helical interfaces are necessary for TIR-TIR self-association, and, subsequent activation of the hypersensitive response. The DE interface was first revealed by the crystal structure of the flax L6 TIR domain (Bernoux et al., 2011). The RRS1 and RPS4 TIR heterodimer crystal indicated TIR-TIR contacts at the AE interfaces, while the RPP1 crystal revealed both AE and DE contacts (Williams et al., 2014; Zhang et al., 2017). Plant TIR-domains vary in strength of TIR-TIR self-associations and in some cases, self-association strength correlates with function (Schreiber et al., 2016; Zhang et al., 2017). The TIR-only protein, RBA1 (Response to HopBA1), self-associates using both AE and DE interfaces (Nishimura et al., 2017). RBA1 self-association is detectable via co-immunoprecipitation or yeast 2-hybrid assay, and both self-association interfaces must be intact to trigger cell death (Nishimura et al., 2017). Similarly, the isolated TIR-domain of the RPV1 TNL is sufficient to activate HR (Williams et al., 2016). However, self-association of RPV1 TIR-domains was not detectable by yeast two-hybrid analysis or size exclusion chromatography (Williams et al., 2016), yet disruption of the AE interface did abolish RPV1-mediated HR (Williams et al., 2016). Thus, intact TIR-TIR interfaces appear necessary for TIR-immune function, and can vary in strength. Additionally, the NBS-LRR domains of modular TNLs also promote oligomerization, and whether TIR-only proteins must evolve stronger TIR-TIR interfaces due to lack of NBS-LRR mediated organization is unclear.

**FIGURE 7:**
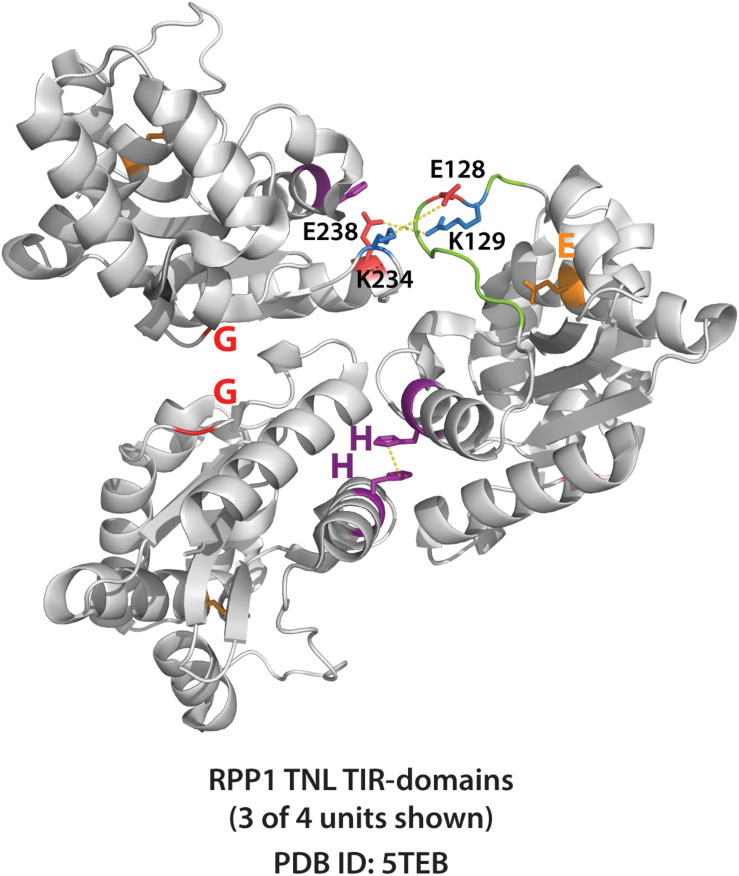
Crystal structure of RPP1 plant TIR-domains showing TIR-TIR interfaces. Three of four RPP1 TIR-domain units shown (PDB ID: 5TEB). The AE (SH 108-109) and DE interfaces (G 229) are shown purple and red, respectively, while putative catalytic glutamate E164 shown orange. Connecting loop above putative catalytic glutamate is colored green. Potential loop interactions between putative ionic pairs of adjacent RPP1 monomers (residues E128 – K234, and R129 – E238) shown with dashed yellow lines. Distances between putative ionic pairs measured using Pymol: E128 to K234 (8.0 Å) and R129 to E238 (4.8 Å).

Similar to cell death and disease resistance phenotypes, the activation of plant TIR NADase function requires both AE and DE self-association interfaces (Horsefield et al., 2019; Wan et al., 2019). It seems likely that the NADase activity of plant TIRs is dependent on some higher-order oligomer that has simultaneously engaged both AE and DE interfaces. Intriguingly, the RPP1 crystal structure (Figure 7) suggests that a loop that covers the catalytic glutamate could play this role, as it is positioned near a neighboring monomer only once both interfaces are engaged. Whether or not crystal structures of isolated TIR domains reflect the orientation in the activated TNL context remains to be determined. Currently, no structure of a full length TNL is available, and thus, how TNL oligomerization mediated by the NBS domains influences TIR–TIR associations, remains unclear. The activation of NADase activity following higher-order TIR oligomerization seems consistent with the behavior of the RBA1 E86A putative catalytic mutant (Wan et al., 2019). RBA1 E86A still self-associates (as measured by co-immunoprecipitation), suggesting that activation of NAD^+^-hydrolysis follows the self-association of TIR-domains.

## Oligomeric Plant “Resistosomes”

The N-terminal coiled coil (CC) domain of some CC-domain type NLRs (e.g., Sr71, NRG1, MLA) can induce HR (Collier et al., 2011; Bai et al., 2012; Casey et al., 2016). Modeling of RPW8-type CC-domains suggests that they may adopt a 4-helix bundle fold similar to that of the mixed-lineage kinase-like protein family of animals, which insert into host membranes and promote cell death (Jubic et al., 2019). Recently, cryo-EM structures for active (ATP-bound) and inactive (ADP-bound) ZAR1 ‘resistosomes’ were determined (Wang et al., 2019a, b). The ZAR1 (HOPZ-ACTIVATED RESISTANCE 1) resistosome complex forms a ring-shaped pentameric structure, and contains bound RKS1 pseudokinase, and an effector-modified kinase, PBL2. The pentameric resistosome structure is driven by the ZAR1 NBS-LRR domains, however, the presence of associated host guardee and adaptor proteins (e.g., RKS1, PBL2) will also influence overall resistosome structure (Wang et al., 2019a). The N-terminal CC-domains of ZAR1 subunits undergo a conformational change, each extending a helix to form a funnel-like structure, which is hypothesized to disrupt membrane integrity and promote cell death (Wang et al., 2019a).

Can the pentameric structure of the ZAR1 resistosome – a CC-domain type NLR - inform what higher order complexes an activated TNL might form? It is enticing to speculate that, like ZAR1 and animal NLRs, an oligomeric TNL NADase complex also forms a ring-shaped resistosome? A variety of stoichiometries are observed for the animal NLR oligomers that form the apoptosome and inflammosome rings (Zhang et al., 2015). The hypothetical TNL resistosome could be of a range of stoichiometries, and most likely forms a ring. However, given the existing structures of plant TIR domains, it seems difficult to reconcile the radial (head to tail) symmetry of a ring-shaped resistosome, no matter the stoichiometry. In these structures, the AE and DE interfaces are in a “head to head” orientation that seems at odds with a circular chain. Perhaps an increase in local concentration of TIR domains is sufficient to promote signaling. Or possibly, these interfaces will not be seen in the context of a full-length TNL oligomer structure. Fusion of the SARM1 SAM domains to either the N-terminus (Horsefield et al., 2019; Wan et al., 2019) or C-terminus (unpublished) of plant TIR-domains enables NADase activity and HR-induction. The SAM domains of SARM1 form an octameric ring (Figure 5B) (Horsefield et al., 2019; Sporny et al., 2019). Even in the context of a fusion protein with forced oligomerization, the RPS4 SAM:TIR protein still requires both AE and DE interfaces (Wan et al., 2019). These results suggest that an octameric ring structure can accommodate plant TIR function, and also that there is surprising flexibility in how functional TIR domain oligomerization can be promoted.

**FIGURE 8:**
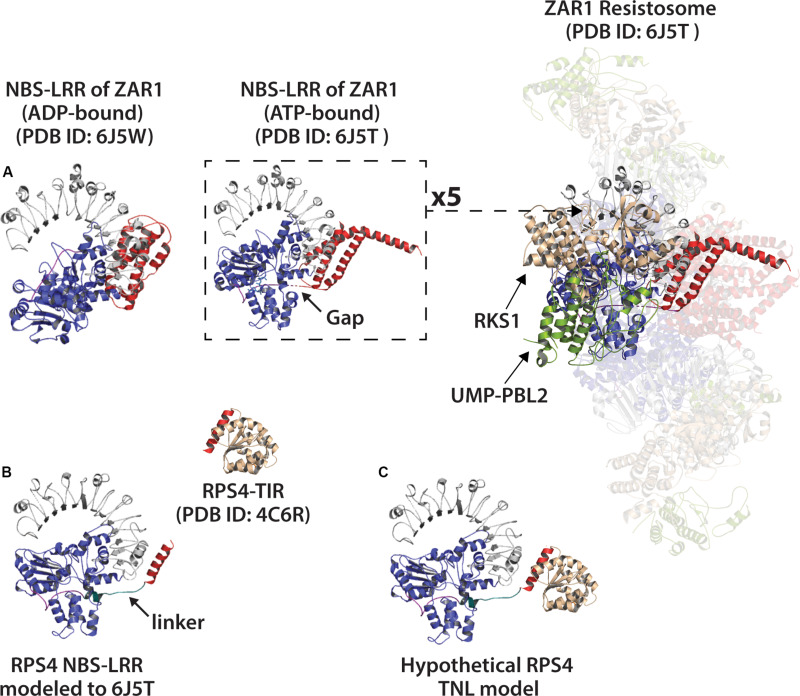
Modeling of the RPS4 NBS-LRR (a TNL) to the NBS-LRR of ZAR1 resistosome (ATP-bound) or ZAR1 monomer (ADP-bound). **(A)** Left: ADP-bound ZAR1 monomer structure as determined by Wang et al. (2019a). Center: single ATP-bound ZAR1 (CNL) subunit from the cryo-EM determined resistosome structure by Wang et al. (2019b). Right: Activated ZAR1-resistosome. Coiled coil (CC) domain of ZAR1 colored red. NBS (nucleotide binding site) colored blue and LRR (leucine rich repeat) colored gray. ZAR1 N-terminal linker regions colored purple, and gaps in linker indicated by arrow. Resistosome-associated proteins RKS1 and effector-modified UMP-PBL2 shown tan and green, respectively. **(B)** Left: Phyre2 modeling of the RPS4 NBS-LRR (including final helix of RPS4 TIR-domain shown in red) to ATP-bound NBS-LRR of the ZAR1 resistosome (PDB ID: 6J5T). The putative RPS4 linker is colored teal and indicated with arrow. Above and right: crystal structure of RPS4 (TNL) TIR-domain (PDB ID: 4C6R) with putative catalytic glutamate (E88) colored orange. **(C)** The RPS4 TIR manually docked onto the RPS4 NBS-LRR model. The red helix shown on RPS4-TIR is the same red helix included in the NBS-LRR model.

Using Phyre2, we modeled the NBS-LRR domains of RPS4 onto the structures of inactive and active ZAR1 NBS-LRRs (Figure 8). The NBS and N-terminal linker regions of RPS4, as compared with ZAR1, are similar in length and potentially in orientation (Figure 8). While entirely speculative, there would appear to be limits on the amount of rotational flexibility the TIR domains would have in a hypothetical resistosome to engage in simultaneous AE and DE interfaces. The oligomerization state of so-called “paired NLRs” – where individual partners typically assume a ‘sensor’ or ‘signal’ role – may be even more complex. Given that RPS4 and RRS1 appear to function in a complex (Huh et al., 2017), what would the stoichiometry and organization of a hetero-oligomeric resistosome look like? The fact that the RRS1 TIR lacks a catalytic glutamate makes the situation even more interesting.

Plant TIR-only proteins can signal despite their lack of C-terminal NBS-LRR domains (Nishimura et al., 2017; Wan et al., 2019). In the absence of oligomerizing NBS-LRR domains, what higher order structures might naturally occurring TIR-only proteins form? The TIR-only protein, RBA1, self-associates and requires the conserved AE and DE-type interfaces. Are TIR-only oligomers different than TNL oligomers? RBA1 also requires EDS1 and NRG1, but like TNL receptors there is still no clear mechanistic link between TIR activation and downstream signal transduction (Nishimura et al., 2017; Wan et al., 2019).

## How Might Plant TIR-NADases Transmit Immune Signals?

NAD^+^ is a major cellular metabolite, redox carrier, and substrate for numerous processes including DNA repair, epigenetic modifications, immunity and signaling (Adams-Phillips et al., 2010; Petriacq et al., 2013; Petriacq et al., 2016). Activated plant TIR-domains are NAD^+^-hydrolases, but how might NAD^+^-consumption activate immune responses? SARM1 apparently triggers cell death by depleting NAD^+^, but plant TIRs do not cause detectable NAD^+^ reductions *in planta* (Wan et al., 2019). One possibility is that NAD^+^ consumption by plant TIRs generates signal molecules that turn on downstream immune components.

Unlike SARM1, plant TIRs did not generate c-ADPR, but instead produced v-cADPR, both *in vitro* and after transient expression in *N. benthamiana* (Wan et al., 2019). Moreover, v-cADPR was also produced by activation of RBA1 after bacterial delivery of the *Pseudomonas syringae* effector HopBA1 (Wan et al., 2019). Neither EDS1 or NRG1 – downstream TIR-signaling components - were required for v-cADPR generation by activated TIRs *in planta* (Wan et al., 2019). These results indicate that v-cADPR accumulation is upstream of both cell death and the known signaling components downstream of TIR proteins. Curiously, the *in planta* generation of v-cADPR by TIR-domains isolated from TNLs was nearly 100-fold lower than that of TIR-only proteins (Wan et al., 2019). Is this difference an artifact of truncating TNL proteins, or an intrinsic difference between TIR-only and TNL TIR-domains? Whether an auto-active variant of a full length TNL might produce comparable v-cADPR to TIR-only proteins has not been examined. It is also unclear if the context of a full length NLR could influence the ratio or type of products generated by NAD^+^-hydrolysis, apart from hydrolysis kinetics.

The v-cADPR molecule appears to uniquely identify plant TIR-driven ETI, as MLA10 expression and RPM1 activation (both CNLs) did not elevate v-cADPR (Wan et al., 2019). The chemical structure of v-cADPR is presently unknown, and could vary significantly from cyclic-ADPR. It is possible that v-cADPR shares signaling properties with other NAD^+^-derivatives such as cyclic-ADPR, ADPR, and NAAD (a product of the SARM1-TIR), which are potent Ca^2+^ channel activators (Lee, 2012; Guse, 2015). Numerous studies reveal Ca^2+^ signaling is necessary for plant immunity and HR-driven cell death (Grant et al., 2000; Ma and Berkowitz, 2007, 2011; Marcec et al., 2019). Intriguingly, cyclic-ADPR has been reported to trigger plant defense gene expression, and a calcium channel blocker, lanthanum chloride, prevents plant cell death and HR (although this is not specific to TIR phenotypes) (Durner et al., 1998; Grant et al., 2000).

At this point v-cADPR can be considered a biomarker for plant TIR activity, as its production is correlated with TIR function, however, it is not clear if it is either necessary or sufficient to trigger cell death or disease resistance. *In vitro* assays indicate that the TIR-only proteins RBA1 and BdTIR are also capable of cleaving NADP^+^ (Wan et al., 2019), and it remains to be determined what the putative v-cADPRP product looks like and if it is produced *in planta*. Are there other, as yet, unidentified products? How NADase-produced signaling products might activate immune responses is entirely speculative, but a reasonable candidate to receive a signal would be EDS1, potentially mediated by an EDS1 hetero-oligomer surface. The fact that EDS1/SAG101 and EDS1/PAD4 heterodimers can have non-redundant functions, with specificity in regards to the particular activating TIR (Cui et al., 2017; Castel et al., 2019; Lapin et al., 2019; Wu et al., 2019), complicate simple models where TIR proteins generate a generic signal.

Because NAD^+^ levels influence numerous cellular processes, the consumption of NAD^+^ by plant TIRs during immunity could impact myriad cellular responses. For instance, extracellular NAD^+^ (eNAD^+^) is a potent immunostimulatory signal and reducing NAD^+^ levels compromises disease resistance; conversely, eNAD^+^ application can bolster immunity (Zhang and Mou, 2012; Wang et al., 2016; Mou, 2017; Alferez et al., 2018). Likewise, the AvrRxo1 and RipN, virulence-promoting effectors of plant pathogens, can modulate host NAD^+^ homeostasis and defense responses (Schuebel et al., 2016; Shidore et al., 2017; Sun et al., 2019). While total NAD^+^ levels did not obviously change with TIR expression (Wan et al., 2019), it’s possible that localization of NADase activity could have an impact on output.

## TIR-Proteins Across Plant Phylogenies

TIR-domain encoding genes can be found in almost all plant lineages. However, the class and abundance of encoded TIR-proteins can vary widely between species (Collier et al., 2011; Yue et al., 2012; Nandety et al., 2013; Sun et al., 2014; Gao et al., 2018). Particularly, between dicot and monocot plant species, the complement of CNL vs. TNL-type NLRs can vary greatly (Sun et al., 2014; Gao et al., 2018). Canonical TNL-type resistance genes are absent from all examined monocot genomes, as are the TIR-pathway mediators, SAG101 and NRG1 (Collier et al., 2011; Wagner et al., 2013). Remarkably, convergent loss of TNLs and downstream genes has occurred several times during plant evolution (Collier et al., 2011; Baggs et al., 2019). Monocots do, however, encode several TIR-NBS and TIR-only genes, although in low abundance relative to the high number of TNLs commonly present in dicots (Sun et al., 2014; Gao et al., 2018). Whether or not these monocot TIR proteins are functioning as immune receptors remains to be determined. However, the TIR-only protein RBA1, can trigger cell death in response a specific pathogen effector, and both TIR-NBS and TIR-X proteins from various plant species are reported to enhance immunity (Meyers et al., 2002; Staal et al., 2008; Nandety et al., 2013; Zhao et al., 2015; Nishimura et al., 2017; Chen et al., 2018; Santamaria et al., 2019). Thus, while TNLs may be absent from monocot genomes, TIR-signaling could play roles in regulating physiological responses and immunity in monocots. BdTIR, a TIR-only protein from the monocot *Brachypodium*, has many of the hallmarks of dicot TIR domains: it has the conserved putative catalytic glutamic acid, produces v-cADPR and triggers EDS1-dependent cell death in *N. benthamiana* (Wan et al., 2019). Intriguingly, BdTIR cell death in *N. benthamiana* is also dependent on the downstream TIR signaling component NbNRG1, despite the fact that monocots have lost *NRG1* from their genomes. Therefore, it is possible that TIR-domains from distant plant phylogenies produce common signals from NAD^+^-hydrolysis, while the putative immune output depends on which downstream components (e.g., EDS1-members, NRG1) are present to enact the signal.

While TNLs are absent from monocots (and several dicot lineages), they are present broadly across the plant phylogeny, including bryophytes and conifers (see also Figure 2C) (Baggs et al., 2019). For instance, the moss *Physcomitrella patens* carries TNL loci, as does the western white pine, *Pinus monticola* (Liu and Ekramoddoullah, 2011; Tanigaki et al., 2014). Two pine TNL loci, TNL1 and TNL2, are correlated with blister rust resistance (Liu and Ekramoddoullah, 2011). TIR-domain-encoding genes were more recently reported in the agriculturally important red algae, *Pyropia yezoensis*, which is used for nori production (Tang et al., 2019). At least one TIR-domain encoding gene, along with several NBS genes of *Pyropia* are upregulated by challenge with the oomycete pathogen, *Pythium* (Tang et al., 2019). Genes with TIR immune receptor-like domain combinations have been found in the genomes of green algae. *Botryococcus* contains TIR-NBS encoding genes, while remarkably, *Chromochloris* has NLR-like genes that contain all three canonical NLR domains (TIR, NBS and LRR) (Shao et al., 2019). More functional evidence for algal TIRs or TNLs in immunity is needed, as well as investigation into the algal relatives of downstream TIR pathway components defined in dicots. It seems likely that TIR-domains across photosynthetic organisms harbor NADase activities, however, this has not been explored. Nor is it clear if these TIR-domains could produce similar molecules from NAD^+^-hydrolysis. An expanded collection of genomic data from algae and early plant clades will help to assess both the conservation and abundance of putative TIR-immune pathways.

## (More) Unanswered Questions?

TIR-domains encoded by species from all domains of life are now known to play roles in immunity. Recent studies now suggest a new paradigm of TIR-mediated immunity in plants: the oligomerization and self-association of TIR-domains, and subsequent hydrolysis of NAD^+^ (Zhang et al., 2017; Horsefield et al., 2019; Wan et al., 2019). Many important and intriguing questions about TIR-immunity remain. For instance, the stoichiometry and confirmation of active plant TNL or TIR-immune complexes is not known. Furthermore, does the NADase activity of plant TIRs generate immunomodulatory signals? And if so, how are these signals transduced and decoded? Finally, the extent of plant TIR functional conservation is not fully known; i.e., are the TIR-domains encoded by more distantly-related photosynthetic lineages also NADases and do they function in or outside of immunity?

If plant TIRs generate immunomodulatory signals from the hydrolysis of NAD(P)^+^, then what is that signal? For instance, might variant-cADPR *per se* be sufficient to activate transcriptional defenses, or the hypersensitivity response? Or might different TIR-derived signal molecules communicate different outputs? Additionally, plant TIR-NADases could potentially regulate NAD^+^ levels and cellular metabolism apart from immune signal generation. Do TIR-domains from all plant lineages generate the same type(s) of signals, and how has evolution shaped the components which sense and translate outputs from these signals? The subcellular localization and expression of both signal generating TIRs, and downstream signal receivers could influence potential response outcomes.

TIR-based immunity appears to have an ancient role in prokaryotes as an anti-viral defense system (Doron et al., 2018; Cohen et al., 2019). The conservation of NADase activity among animal, plant and prokaryotic TIRs suggests that an ancient enzymatic activity has been re-purposed multiple times in eukaryotic evolution to promote cell death or immune function. A particularly intriguing question is how did plant TNLs and TIRs evolve to become reliant on the downstream EDS1-family and ‘helper’ NLR partners? Presumably, these components independently provided host benefits, prior to co-evolution into overlapping networks. An in-depth analysis of genomes from early plant lineages may provide insights into how TIRs, EDS1-members and ‘helper’ NLRs co-evolved to function in a core pathway, and provide clues into the mechanisms of TIR-signaling networks of higher plants.

Combined biochemical and evolutionary approaches may provide guidance into how variation in the TIR active site or TIR association interfaces could affect immune outputs. In the future, such findings may be able to offer predictions regarding the kinetic properties of specific TIR-domains, as well as a likely profile of NAD^+^-derived products. For instance, might modulating NAD^+^-hydrolysis kinetics and/or product profile influence the type or strength of immune output? Can *in vitro* evolution enable ‘tweaking’ of TIR-active sites, or of TIR-TIR self-association interfaces, and thus alter the profile of products derived from NAD^+^-hydrolysis?

The recognition that TIR domains across the tree of life have conserved enzymatic functions has opened new avenues of investigation into the plant immune system. While much remains undiscovered, the field is poised to describe fully connected NLR signaling pathways that lead to immune outputs. This synthesis will enable rational engineering of plant immunity to help address the increasing demands on our agricultural systems.

## Author Contributions

AB and MN wrote the manuscript. Both authors approved the manuscript.

## Conflict of Interest

The authors declare that the research was conducted in the absence of any commercial or financial relationships that could be construed as a potential conflict of interest.

## References

[B1] Adams-Phillips L. Briggs A. G. Bent A. F. (2010). Disruption of poly(ADP-ribosyl)ation mechanisms alters responses of *Arabidopsis* to biotic stress. *Plant Physiol.* 152 267–280. 10.1104/pp.109.148049 19889874 PMC2799362

[B2] Alaidarous M. Ve T. Casey L. W. Valkov E. Ericsson D. J. Ullah M. O. (2014). Mechanism of bacterial interference with TLR4 signaling by *Brucella* Toll/interleukin-1 receptor domain-containing protein TcpB. *J. Biol. Chem.* 289 654–668. 10.1074/jbc.M113.523274 24265315 PMC3887194

[B3] Alferez F. M. Gerberich K. M. Li J. L. Zhang Y. Graham J. H. Mou Z. (2018). Exogenous nicotinamide adenine dinucleotide induces resistance to citrus canker in citrus. *Front. Plant Sci.* 9:1472. 10.3389/fpls.2018.01472 30356715 PMC6189366

[B4] Baggs E. Thanki A. O’Grady R. Schudoma C. Haerty W. Krasileva K. (2019). Convergent loss of an EDS1/PAD4 signalling pathway in several plant lineages predicts new components of plant immunity and drought response. *bioRxiv* [preprint]. 10.1101/572560 PMC7346574 32409319

[B5] Bai S. Liu J. Chang C. Zhang L. Maekawa T. Wang Q. (2012). Structure-function analysis of barley NLR immune receptor MLA10 reveals its cell compartment specific activity in cell death and disease resistance. *PLoS Pathog* 8:e1002752. 10.1371/journal.ppat.1002752 22685408 PMC3369952

[B6] Bernoux M. Ve T. Williams S. Warren C. Hatters D. Valkov E. (2011). Structural and functional analysis of a plant resistance protein TIR domain reveals interfaces for self-association, signaling, and autoregulation. *Cell Host Microbe* 9 200–211. 10.1016/j.chom.2011.02.009 21402359 PMC3142617

[B7] Bhandari D. D. Lapin D. Kracher B. von Born P. Bautor J. Niefind K. (2019). An EDS1 heterodimer signalling surface enforces timely reprogramming of immunity genes in *Arabidopsis*. *Nat. Commun.* 10 772. 10.1038/s41467-019-08783-0 30770836 PMC6377607

[B8] Boutrot F. Zipfel C. (2017). Function, discovery, and exploitation of plant pattern recognition receptors for broad-spectrum disease resistance. *Annu. Rev. Phytopathol.* 55 257–286. 10.1146/annurev-phyto-080614-120106 28617654

[B9] Bovijn C. Ulrichts P. De Smet A. S. Catteeuw D. Beyaert R. Tavernier J. (2012). Identification of interaction sites for dimerization and adapter recruitment in Toll/interleukin-1 receptor (TIR) domain of Toll-like receptor 4. *J. Biol. Chem.* 287 4088–4098. 10.1074/jbc.M111.282350 22139835 PMC3281722

[B10] Casey L. W. Lavrencic P. Bentham A. R. Cesari S. Ericsson D. J. Croll T. (2016). The CC domain structure from the wheat stem rust resistance protein Sr33 challenges paradigms for dimerization in plant NLR proteins. *Proc. Natl. Acad. Sci. U.S.A.* 113 12856–12861. 10.1073/pnas.1609922113 27791121 PMC5111715

[B11] Castel B. Ngou P. M. Cevik V. Redkar A. Kim D. S. Yang Y. (2019). Diverse NLR immune receptors activate defence via the RPW8-NLR NRG1. *New Phytol.* 222 966–980. 10.1111/nph.15659 30582759

[B12] Chan S. L. Low L. Y. Hsu S. Li S. Liu T. Santelli E. (2009). Molecular mimicry in innate immunity: crystal structure of a bacterial TIR domain. *J. Biol. Chem.* 284 21386–21392. 10.1074/jbc.C109.007591 19535337 PMC2755863

[B13] Chen G. Wei B. Li G. Gong C. Fan R. Zhang X. (2018). TaEDS1 genes positively regulate resistance to powdery mildew in wheat. *Plant Mol. Biol.* 96 607–625. 10.1007/s11103-018-0718-9 29582247

[B14] Cohen D. Melamed S. Millman A. Shulman G. Oppenheimer-Shaanan Y. Kacen A. (2019). Cyclic GMP-AMP signalling protects bacteria against viral infection. *Nature* 574 691–695. 10.1038/s41586-019-1605-5 31533127

[B15] Collier S. M. Hamel L. P. Moffett P. (2011). Cell death mediated by the N-terminal domains of a unique and highly conserved class of NB-LRR protein. *Mol. Plant Microbe Interact.* 24 918–931. 10.1094/MPMI-03-11-0050 21501087

[B16] Coronas-Serna J. M. Louche A. Rodríguez-Escudero M. Roussin M. Imbert P. R. C. Rodríguez-Escudero I. (2019). The TIR-domain containing effectors BtpA and BtpB from Brucella abortus block energy metabolism. *bioRxiv* [Preprint]. 10.1101/703330 PMC7188309 32298382

[B17] Cui H. Gobbato E. Kracher B. Qiu J. Bautor J. Parker J. E. (2017). A core function of EDS1 with PAD4 is to protect the salicylic acid defense sector in Arabidopsis immunity. *New Phytol.* 213 1802–1817. 10.1111/nph.14302 27861989

[B18] Dangl J. L. Horvath D. M. Staskawicz B. J. (2013). Pivoting the plant immune system from dissection to deployment. *Science* 341 746–751. 10.1126/science.1236011 23950531 PMC3869199

[B19] Doron S. Melamed S. Ofir G. Leavitt A. Lopatina A. Keren M. (2018). Systematic discovery of antiphage defense systems in the microbial pangenome. *Science* 359:eaar4120. 10.1126/science.aar4120 29371424 PMC6387622

[B20] Durner J. Wendehenne D. Klessig D. F. (1998). Defense gene induction in tobacco by nitric oxide, cyclic GMP, and cyclic ADP-ribose. *Proc. Natl. Acad. Sci. U.S.A.* 95 10328–10333. 10.1073/pnas.95.17.10328 9707647 PMC21508

[B21] Essuman K. Summers D. W. Sasaki Y. Mao X. DiAntonio A. Milbrandt J. (2017). The SARM1 Toll/Interleukin-1 receptor domain possesses intrinsic NAD(+) cleavage activity that promotes pathological axonal degeneration. *Neuron* 93 1334–1343.e5. 10.1016/j.neuron.2017.02.022 28334607 PMC6284238

[B22] Essuman K. Summers D. W. Sasaki Y. Mao X. Yim A. K. Y. DiAntonio A. (2018). TIR domain proteins are an ancient family of NAD(+)-consuming enzymes. *Curr. Biol.* 28 421430.e4. 10.1016/j.cub.2017.12.024 29395922 PMC5802418

[B23] Feys B. J. Wiermer M. Bhat R. A. Moisan L. J. Medina-Escobar N. Neu C. (2005). Arabidopsis SENESCENCE-ASSOCIATED GENE101 stabilizes and signals within an ENHANCED DISEASE SUSCEPTIBILITY1 complex in plant innate immunity. *Plant Cell.* 17 2601–2613. 10.1105/tpc.105.033910 16040633 PMC1197438

[B24] Gao Y. Wang W. Zhang T. Gong Z. Zhao H. Han G. Z. (2018). Out of water: the origin and early diversification of plant R-Genes. *Plant Physiol.* 177 82–89. 10.1104/pp.18.00185 29563207 PMC5933115

[B25] Gerdts J. Brace E. J. Sasaki Y. DiAntonio A. Milbrandt J. (2015). SARM1 activation triggers axon degeneration locally via NAD(+) destruction. *Science* 348 453–457. 10.1126/science.1258366 25908823 PMC4513950

[B26] Grant M. Brown I. Adams S. Knight M. Ainslie A. Mansfield J. (2000). The RPM1 plant disease resistance gene facilitates a rapid and sustained increase in cytosolic calcium that is necessary for the oxidative burst and hypersensitive cell death. *Plant J.* 23 441–450. 10.1046/j.1365-313x.2000.00804.x 10972870

[B27] Guse A. H. (2015). Calcium mobilizing second messengers derived from NAD. *Biochim. Biophys. Acta* 1854 1132–1137. 10.1016/j.bbapap.2014.12.015 25534250

[B28] Heidrich K. Wirthmueller L. Tasset C. Pouzet C. Deslandes L. Parker J. E. (2011). Arabidopsis EDS1 connects pathogen effector recognition to cell compartment-specific immune responses. *Science* 334 1401–1404. 10.1126/science.1211641 22158818

[B29] Horsefield S. Burdett H. Zhang X. Manik M. K. Shi Y. Chen J. (2019). NAD(+) cleavage activity by animal and plant TIR domains in cell death pathways. *Science* 365 793–799. 10.1126/science.aax1911 31439792

[B30] Hu Z. Zhou Q. Zhang C. Fan S. Cheng W. Zhao Y. (2015). Structural and biochemical basis for induced self-propagation of NLRC4. *Science* 350 399–404. 10.1126/science.aac5489 26449475

[B31] Huh S. U. Cevik V. Ding P. Duxbury Z. Ma Y. Tomlinson L. (2017). Protein-protein interactions in the RPS4/RRS1 immune receptor complex. *PLoS Pathog* 13:e1006376. 10.1371/journal.ppat.1006376 28475615 PMC5435354

[B32] Jones J. D. Dangl J. L. (2006). The plant immune system. *Nature* 444 323–329. 17108957 10.1038/nature05286

[B33] Jubic L. M. Saile S. Furzer O. J. El Kasmi F. Dangl J. L. (2019). Help wanted: helper NLRs and plant immune responses. *Curr. Opin. Plant Biol.* 50 82–94. 10.1016/j.pbi.2019.03.013 31063902

[B34] Kenny E. F. O’Neill L. A. (2008). Signalling adaptors used by Toll-like receptors: an update. *Cytokine* 43 342–349. 10.1016/j.cyto.2008.07.010 18706831

[B35] Lapin D. Kovacova V. Sun X. Dongus J. A. Bhandari D. D. von Born P. (2019). A coevolved EDS1-SAG101-NRG1 module mediates cell death signaling by TIR-domain immune receptors. *Plant Cell* 31 24302455, 10.1105/tpc.19.00118 31311833 PMC6790079

[B36] Lee H. C. (2012). Cyclic ADP-ribose and nicotinic acid adenine dinucleotide phosphate (NAADP) as messengers for calcium mobilization. *J. Biol. Chem.* 287 31633–31640. 10.1074/jbc.R112.349464 22822066 PMC3442497

[B37] Lee H. C. Zhao Y. J. (2019). Resolving the topological enigma in Ca(2+) signaling by cyclic ADP-ribose and NAADP. *J. Biol. Chem.* 294 19831–19843. 10.1074/jbc.REV119.009635 31672920 PMC6937575

[B38] Liu J. J. Ekramoddoullah A. K. (2011). Genomic organization, induced expression and promoter activity of a resistance gene analog (PmTNL1) in western white pine (Pinus monticola). *Planta* 233 1041–1053. 10.1007/s00425-011-1353-8 21279649

[B39] Loring H. S. Icso J. D. Nemmara V. Thompson P. R. (2020). Initial kinetic characterization of sterile alpha and Toll/Interleukin receptor motif-containing protein 1. *Biochemistry* 59 933942. 10.1021/acs.biochem.9b01078 32049506 PMC7085114

[B40] Ma W. Berkowitz G. A. (2007). The grateful dead: calcium and cell death in plant innate immunity. *Cell Microbiol.* 9 2571–2585. 10.1111/j.1462-5822.2007.01031.x 17714518

[B41] Ma W. Berkowitz G. A. (2011). Ca2+ conduction by plant cyclic nucleotide gated channels and associated signaling components in pathogen defense signal transduction cascades. *New Phytol.* 190 566–572. 10.1111/j.1469-8137.2010.03577.x 21166809

[B42] Marcec M. J. Gilroy S. Poovaiah B. W. Tanaka K. (2019). Mutual interplay of Ca(2+) and ROS signaling in plant immune response. *Plant Sci.* 283 343–354. 10.1016/j.plantsci.2019.03.004 31128705

[B43] Meyers B. C. Morgante M. Michelmore R. W. (2002). TIR-X and TIR-NBS proteins: two new families related to disease resistance TIR-NBS-LRR proteins encoded in *Arabidopsis* and other plant genomes. *Plant J.* 32 77–92. 10.1046/j.1365-313x.2002.01404.x 12366802

[B44] Mou Z. (2017). Extracellular pyridine nucleotides as immune elicitors in arabidopsis. *Plant Signal. Behav.* 12:e1388977. 10.1080/15592324.2017.1388977 29035673 PMC5703255

[B45] Murata H. Khine C. C. Nishikawa A. Yamamoto K. I. Kinoshita R. Sakaguchi M. (2018). c-Jun N-terminal kinase (JNK)-mediated phosphorylation of SARM1 regulates NAD(+) cleavage activity to inhibit mitochondrial respiration. *J. Biol. Chem.* 293 18933–18943. 10.1074/jbc.RA118.004578 30333228 PMC6295714

[B46] Nandety R. S. Caplan J. L. Cavanaugh K. Perroud B. Wroblewski T. Michelmore R. W. (2013). The role of TIR-NBS and TIR-X proteins in plant basal defense responses. *Plant Physiol.* 162 1459–1472. 10.1104/pp.113.219162 23735504 PMC3707564

[B47] Nanson J. D. Kobe B. Ve T. (2019). Death, TIR, and RHIM: self-assembling domains involved in innate immunity and cell-death signaling. *J. Leukoc. Biol.* 105 363–375. 10.1002/JLB.MR0318-123R 30517972

[B48] Nimma S. Ve T. Williams S. J. Kobe B. (2017). Towards the structure of the TIR-domain signalosome. *Curr. Opin. Struct. Biol.* 43 122–130. 10.1016/j.sbi.2016.12.014 28092811

[B49] Nishimura M. T. Anderson R. G. Cherkis K. A. Law T. F. Liu Q. L. Machius M. (2017). TIR-only protein RBA1 recognizes a pathogen effector to regulate cell death in *Arabidopsis*. *Proc. Natl. Acad. Sci. U.S.A.* 114 E2053–E2062. 10.1073/pnas.1620973114 28137883 PMC5347586

[B50] Nyman T. Stenmark P. Flodin S. Johansson I. Hammarstrom M. Nordlund P. (2008). The crystal structure of the human toll-like receptor 10 cytoplasmic domain reveals a putative signaling dimer. *J. Biol. Chem.* 283 11861–11865. 10.1074/jbc.C800001200 18332149

[B51] O’Neill L. A. Bowie A. G. (2007). The family of five: TIR-domain-containing adaptors in Toll-like receptor signalling. *Nat. Rev. Immunol.* 7 353–364. 10.1038/nri2079 17457343

[B52] Peart J. R. Mestre P. Lu R. Malcuit I. Baulcombe D. C. (2005). NRG1, a CC-NB-LRR protein, together with N, a TIR-NB-LRR protein, mediates resistance against tobacco mosaic virus. *Curr. Biol.* 15 968–973. 10.1016/j.cub.2005.04.053 15916955

[B53] Petriacq P. de Bont L. Tcherkez G. Gakiere B. (2013). NAD: not just a pawn on the board of plant-pathogen interactions. *Plant Signal. Behav.* 8:e22477. 10.4161/psb.22477 23104110 PMC3745554

[B54] Petriacq P. Ton J. Patrit O. Tcherkez G. Gakiere B. (2016). NAD Acts as an Integral Regulator of Multiple Defense Layers. *Plant Physiol.* 172 1465–1479. 10.1104/pp.16.00780 27621425 PMC5100754

[B55] Qi D. Innes R. W. (2013). Recent advances in plant NLR structure, function, localization, and signaling. *Front. Immunol.* 4:348. 10.3389/fimmu.2013.00348 24155748 PMC3801107

[B56] Qi T. Seong K. Thomazella D. P. T. Kim J. R. Pham J. Seo E. (2018). NRG1 functions downstream of EDS1 to regulate TIR-NLR-mediated plant immunity in *Nicotiana benthamiana*. *Proc. Natl. Acad. Sci. U.S.A.* 115 E10979–E10987. 10.1073/pnas.1814856115 30373842 PMC6243234

[B57] Santamaria M. E. Martinez M. Arnaiz A. Rioja C. Burow M. Grbic V. (2019). An *Arabidopsis* TIR-Lectin two-domain protein confers defense properties against *Tetranychus urticae*. *Plant Physiol.* 179 1298–1314. 10.1104/pp.18.00951 30765478 PMC6446783

[B58] Schreiber K. J. Bentham A. Williams S. J. Kobe B. Staskawicz B. J. (2016). Multiple domain associations within the arabidopsis immune receptor RPP1 regulate the activation of programmed cell death. *PLoS Pathog* 12:e1005769 10.1371/journal.ppat.100576 PMC4948778 27427964

[B59] Schuebel F. Rocker A. Edelmann D. Schessner J. Brieke C. Meinhart A. (2016). 3’-NADP and 3’-NAADP, Two Metabolites Formed by the Bacterial Type III Effector AvrRxo1. *J. Biol. Chem.* 291 22868–22880. 10.1074/jbc.M116.751297 27621317 PMC5087710

[B60] Shao Z. Q. Xue J. Y. Wang Q. Wang B. Chen J. Q. (2019). Revisiting the Origin of Plant NBS-LRR Genes. *Trends Plant Sci.* 24 9–12. 10.1016/j.tplants.2018.10.015 30446304

[B61] Shidore T. Broeckling C. D. Kirkwood J. S. Long J. J. Miao J. Zhao B. (2017). The effector AvrRxo1 phosphorylates NAD in planta. *PLoS Pathog* 13:e1006442. 10.1371/journal.ppat.1006442 28628666 PMC5491322

[B62] Shivers R. P. Kooistra T. Chu S. W. Pagano D. J. Kim D. H. (2009). Tissue-specific activities of an immune signaling module regulate physiological responses to pathogenic and nutritional bacteria in C. Elegans. *Cell Host Microbe* 6 321–330. 10.1016/j.chom.2009.09.001 19837372 PMC2772662

[B63] Snyder G. A. Deredge D. Waldhuber A. Fresquez T. Wilkins D. Z. Smith P. T. (2014). Crystal structures of the Toll/Interleukin-1 receptor (TIR) domains from the *Brucella* protein TcpB and host adaptor TIRAP reveal mechanisms of molecular mimicry. *J. Biol. Chem.* 289 669–679. 10.1074/jbc.M113.523407 24275656 PMC3887195

[B64] Spear A. M. Loman N. J. Atkins H. S. Pallen M. J. (2009). Microbial TIR domains: not necessarily agents of subversion? *Trends Microbiol.* 17 393–398. 10.1016/j.tim.2009.06.005 19716705

[B65] Sporny M. Guez-Haddad J. Lebendiker M. Ulisse V. Volf A. Mim C. (2019). Structural evidence for an octameric ring arrangement of SARM1. *J. Mol. Biol.* 431 3591–3605. 10.1016/j.jmb.2019.06.030 31278906

[B66] Staal J. Kaliff M. Dewaele E. Persson M. Dixelius C. (2008). RLM3, a TIR domain encoding gene involved in broad-range immunity of *Arabidopsi*s to necrotrophic fungal pathogens. *Plant J.* 55 188–200. 10.1111/j.1365-313X.2008.03503.x 18397376

[B67] Sun X. Pang H. Li M. Chen J. Hang Y. (2014). Tracing the origin and evolution of plant TIR-encoding genes. *Gene* 546 408–416. 10.1016/j.gene.2014.04.060 24786214

[B68] Sun Y. Li P. Shen D. Wei Q. He J. Lu Y. (2019). The *Ralstonia solanacearum* effector RipN suppresses plant PAMP-triggered immunity, localizes to the endoplasmic reticulum and nucleus, and alters the NADH/NAD(+) ratio in *Arabidopsis*. *Mol. Plant Pathol.* 20 533–546. 10.1111/mpp.12773 30499216 PMC6637912

[B69] Swiderski M. R. Birker D. Jones J. D. (2009). The TIR domain of TIR-NB-LRR resistance proteins is a signaling domain involved in cell death induction. *Mol. Plant Microbe Interact.* 22 157–165. 10.1094/MPMI-22-2-0157 19132868

[B70] Takken F. L. Goverse A. (2012). How to build a pathogen detector: structural basis of NB-LRR function. *Curr. Opin. Plant Biol.* 15 375–384. 10.1016/j.pbi.2012.05.001 22658703

[B71] Tang D. Wang G. Zhou J. M. (2017). Receptor kinases in plant-pathogen interactions: more than pattern recognition. *Plant Cell* 29 618–637. 10.1105/tpc.16.00891 28302675 PMC5435430

[B72] Tang L. Qiu L. Liu C. Du G. Mo Z. Tang X. (2019). Transcriptomic insights into innate immunity responding to red rot disease in red alga *Pyropia yezoensis*. *Int. J. Mol. Sci.* 20:5970. 10.3390/ijms20235970 31783543 PMC6928737

[B73] Tanigaki Y. Ito K. Obuchi Y. Kosaka A. Yamato K. T. Okanami M. (2014). Physcomitrella patens has kinase-LRR R gene homologs and interacting proteins. *PLoS one* 9:e95118. 10.1371/journal.pone.0095118 24748046 PMC3991678

[B74] Urbach J. M. Ausubel F. M. (2017). The NBS-LRR architectures of plant R-proteins and metazoan NLRs evolved in independent events. *Proc. Natl. Acad. Sci. U.S.A.* 114 1063–1068. 10.1073/pnas.1619730114 28096345 PMC5293065

[B75] Valkov E. Stamp A. Dimaio F. Baker D. Verstak B. Roversi P. (2011). Crystal structure of Toll-like receptor adaptor MAL/TIRAP reveals the molecular basis for signal transduction and disease protection. *Proc. Natl. Acad. Sci. U.S.A.* 108 14879–14884. 10.1073/pnas.1104780108 21873236 PMC3169156

[B76] Ve T. Williams S. J. Kobe B. (2015). Structure and function of Toll/interleukin-1 receptor/resistance protein (TIR) domains. *Apoptosis* 20 250–261. 10.1007/s10495-014-1064-2 25451009

[B77] Wagner S. Stuttmann J. Rietz S. Guerois R. Brunstein E. Bautor J. (2013). Structural basis for signaling by exclusive EDS1 heteromeric complexes with SAG101 or PAD4 in plant innate immunity. *Cell Host Microbe* 14 619–630. 10.1016/j.chom.2013.11.006 24331460

[B78] Wan L. Essuman K. Anderson R. G. Sasaki Y. Monteiro F. Chung E. H. (2019). TIR domains of plant immune receptors are NAD(+)-cleaving enzymes that promote cell death. *Science* 365 799–803. 10.1126/science.aax1771 31439793 PMC7045805

[B79] Wang C. Zhang X. Mou Z. (2016). Comparison of nicotinamide adenine dinucleotide phosphate-induced immune responses against biotrophic and necrotrophic pathogens in *Arabidopsis thaliana*. *Plant Signal. Behav.* 11:e1169358. 10.1080/15592324.2016.1169358 27031653 PMC4973797

[B80] Wang J. Hu M. Wang J. Qi J. Han Z. Wang G. (2019a). Reconstitution and structure of a plant NLR resistosome conferring immunity. *Science* 364:eaav5870. 10.1126/science.aav5870 30948527

[B81] Wang J. Wang J. Hu M. Wu S. Qi J. Wang G. (2019b). Ligand-triggered allosteric ADP release primes a plant NLR complex. *Science* 364:eaav5868. 10.1126/science.aav5868 30948526

[B82] Williams S. J. Sohn K. H. Wan L. Bernoux M. Sarris P. F. Segonzac C. (2014). Structural basis for assembly and function of a heterodimeric plant immune receptor. *Science* 344 299–303. 10.1126/science.1247357 24744375

[B83] Williams S. J. Yin L. Foley G. Casey L. W. Outram M. A. Ericsson D. J. (2016). Structure and function of the TIR domain from the grape NLR protein RPV1. *Front. Plant. Sci.* 7:1850. 10.3389/fpls.2016.01850 28008335 PMC5143477

[B84] Wu Z. Li M. Dong O. X. Xia S. Liang W. Bao Y. (2019). Differential regulation of TNL-mediated immune signaling by redundant helper CNLs. *New Phytol.* 222 938–953. 10.1111/nph.15665 30585636

[B85] Xu Y. Tao X. Shen B. Horng T. Medzhitov R. Manley J. L. (2000). Structural basis for signal transduction by the Toll/interleukin-1 receptor domains. *Nature* 408 111–115. 10.1038/35040600 11081518

[B86] Yue J. X. Meyers B. C. Chen J. Q. Tian D. Yang S. (2012). Tracing the origin and evolutionary history of plant nucleotide-binding site-leucine-rich repeat (NBS-LRR) genes. *New Phytol.* 193 1049–1063. 10.1111/j.1469-8137.2011.04006.x 22212278

[B87] Zhang L. Chen S. Ruan J. Wu J. Tong A. B. Yin Q. (2015). Cryo-EM structure of the activated NAIP2-NLRC4 inflammasome reveals nucleated polymerization. *Science* 350 404–409. 10.1126/science.aac5789 26449474 PMC4640189

[B88] Zhang Q. Zmasek C. M. Cai X. Godzik A. (2011). TIR domain-containing adaptor SARM is a late addition to the ongoing microbe-host dialog. *Dev. Comp. Immunol.* 35 461–468. 10.1016/j.dci.2010.11.013 21110998 PMC3085110

[B89] Zhang X. Bernoux M. Bentham A. R. Newman T. E. Ve T. Casey L. W. (2017). Multiple functional self-association interfaces in plant TIR domains. *Proc. Natl. Acad. Sci. U.S.A.* 114 E2046–E2052. 10.1073/pnas.1621248114 28159890 PMC5347627

[B90] Zhang X. Mou Z. (2012). Expression of the human NAD(P)-metabolizing ectoenzyme CD38 compromises systemic acquired resistance in *Arabidopsis*. *Mol. Plant Microbe Interact.* 25 1209–1218. 10.1094/MPMI-10-11-0278 22670756

[B91] Zhao T. Rui L. Li J. Nishimura M. T. Vogel J. P. Liu N. (2015). A truncated NLR protein, TIR-NBS2, is required for activated defense responses in the exo70B1 mutant. *PLoS Genet.* 11:e1004945. 10.1371/journal.pgen.1004945 25617755 PMC4305288

[B92] Zhao Z. Y. Xie X. J. Li W. H. Liu J. Chen Z. Zhang B. (2019). A Cell-Permeant Mimetic of NMN Activates SARM1 to Produce Cyclic ADP-Ribose and Induce Non-apoptotic Cell Death. *iScience* 15 452–466. 10.1016/j.isci.2019.05.001 31128467 PMC6531917

